# Sources of salinity and arsenic in groundwater in southwest Bangladesh

**DOI:** 10.1186/s12932-016-0036-6

**Published:** 2016-07-11

**Authors:** John C. Ayers, Steven Goodbred, Gregory George, David Fry, Laura Benneyworth, George Hornberger, Kushal Roy, Md. Rezaul Karim, Farjana Akter

**Affiliations:** Department of Earth & Environmental Sciences, Vanderbilt University, PMB 351805, 2301 Vanderbilt Place, Nashville, TN 37235-1805 USA; Environmental Science Discipline, Khulna University, Khulna, 9208 Bangladesh; Department of Civil & Environmental Engineering, Vanderbilt University, PMB 351831, 2301 Vanderbilt Place, Nashville, TN 37235-1831 USA

**Keywords:** Salinization, Arsenic, Groundwater, Water chemistry, Bangladesh

## Abstract

**Background:**

High salinity and arsenic (As) concentrations in groundwater are widespread problems in the tidal deltaplain of southwest Bangladesh. To identify the sources of dissolved salts and As, groundwater samples from the regional shallow Holocene aquifer were collected from tubewells during the dry (May) and wet (October) seasons in 2012–2013. Thirteen drill cores were logged and 27 radiocarbon ages measured on wood fragments to characterize subsurface stratigraphy.

**Results:**

Drill cuttings, exposures in pits and regional studies reveal a >5 m thick surface mud cap overlying a ~30 m thick upper unit of interbedded mud and fine sand layers, and a coarser lower unit up to 60 m thick dominated by clean sands, all with significant horizontal variation in bed continuity and thickness. This thick lower unit accreted at rates of ~2 cm/year through the early Holocene, with local subsidence or compaction rates of 1–3 mm/year. Most tubewells are screened at depths of 15–52 m in sediments deposited 8000–9000 YBP. Compositions of groundwater samples from tubewells show high spatial variability, suggesting limited mixing and low and spatially variable recharge rates and flow velocities. Groundwaters are Na–Cl type and predominantly sulfate-reducing, with specific conductivity (SpC) from 3 to 29 mS/cm, high dissolved organic carbon (DOC) 11–57 mg/L and As 2–258 ug/L, and low sulfur (S) 2–33 mg/L.

**Conclusions:**

Groundwater compositions can be explained by burial of tidal channel water and subsequent reaction with dissolved organic matter, resulting in anoxia, hydrous ferric oxide (HFO) reduction, As mobilization, and sulfate (SO_4_) reduction and removal in the shallow aquifer. Introduction of labile organic carbon in the wet season as rice paddy fertilizer may also cause HFO reduction and As mobilization. Variable modern recharge occurred in areas where the clay cap pinches out or is breached by tidal channels, which would explain previously measured ^14^C groundwater ages being less than depositional ages. Of samples collected from the shallow aquifer, Bangladesh Government guidelines are exceeded in 46 % for As and 100 % for salinity.

## Background

This study concerns groundwater resources in a polder in the coastal zone of southwest Bangladesh, where islands built from river sediment are surrounded by tidal channels containing seasonally fresh to brackish water. The objectives of this study are to characterize concentrations of dissolved salts and arsenic in groundwater and to identify their sources.

### Geologic history, the land surface and subsurface stratigraphy

**Fig. 1:**
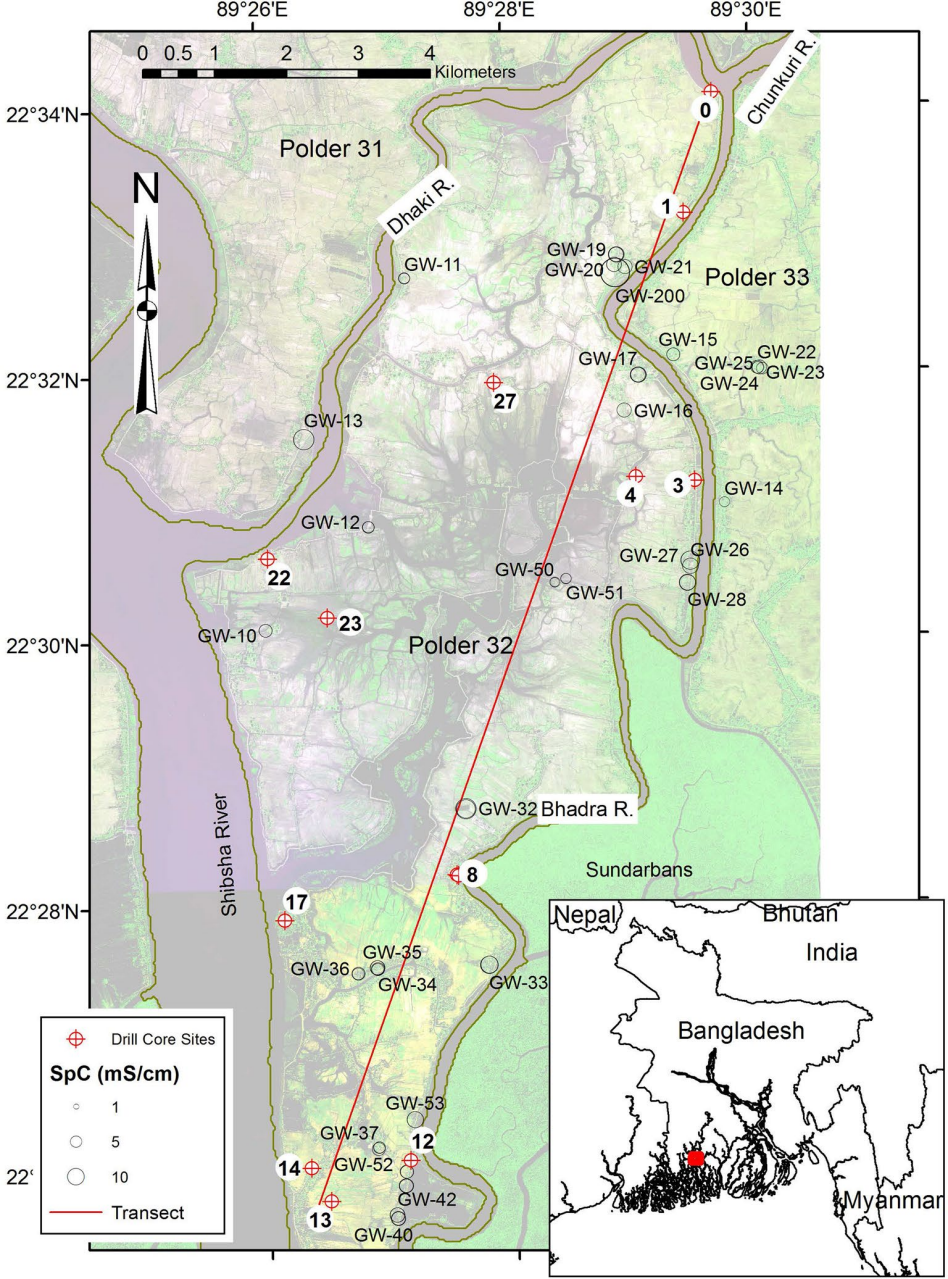
Locations of drill core and tubewell sampling sites. The *red square* in the *inset map* shows the regional location of the larger map. Background is a composite 2012 GeoEye satellite image. Tubewell symbol size is proportional to average measured specific conductivity of groundwater samples collected from each tubewell. The *red line* is the section to which sampled wells and drill core sites are projected to create the profile in Fig. 2

Field work focused on Polder 32 in Khulna district, Dacope upazila, about 30 km south of the city of Khulna and about 60 km north of the Bay of Bengal (Fig. 1). This area is referred to as the South-western Ganges Tidal Floodplain [1]. It experiences a humid, biseasonal climate with a dry season from November to May and wet season from June to October [2]. The polder is 19.3 km by 7.1 km with a total area of 68.2 km^2^ and a population of roughly 40,000. It is bounded by tidal channels including the Dhaki River in the north and northwest, the Bhadra River in the southeast, and the Shibsha River in the west and southwest (Fig. 1). The Sundarbans, a protected mangrove forest, lies to the SE and SW of the polder. Polder 32 was likely part of the mangrove forest before deforestation in the 18th century A.D. (~250 YBP), and polder embankments were constructed in the 1960s and 1970s.

The study area is in a part of the Ganges-Brahmaputra-Meghna delta that currently has a relatively stable elevation due to subsidence rates and accretion rates being comparable [3, 4]. Phases of delta construction during late Quaternary highstands of sea level have formed three principal aquifers in SW Bangladesh, each composed of medium to fine sands capped by fine-grained aquitards from the deposition of tidal or overbank silts [5]. Grey, reduced Holocene sediments containing abundant organic matter generally overlay Pleistocene sediments, that in interfluve regions have been weathered and oxidized to form distinct paleosols. Generally low groundwater As concentrations are found in the Pleistocene aquifers, while the Holocene aquifer generally has high As and Fe concentrations [6, 7].

**Fig. 2:**
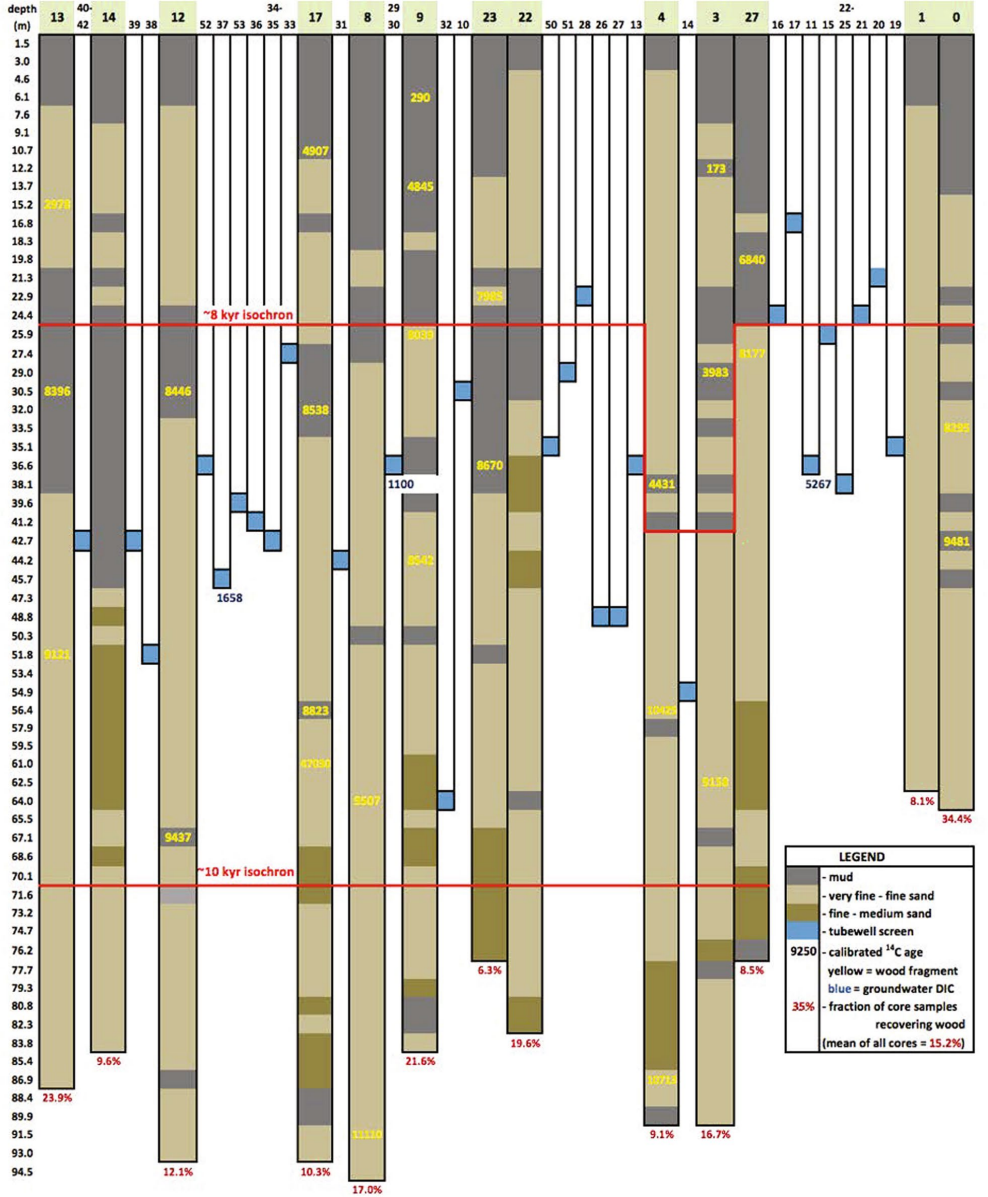
Summary of sediment lithology and radiocarbon ages from the thirteen Polder 32 cores. Drill core and tubewell sampling sites were projected to the* red profile* line in Fig. 1. *Yellow numbers* are calibrated radiocarbon ages in calendar years before present of mangrove wood recovered from the cores. *Horizontal lines* mark the approximate locations of the 8000 and 10,000 year isochrons. *Numbers* beneath tubewells are ^14^C groundwater ages [11]

On Polder 32, tubewells only penetrate into the shallow Holocene aquifer that extends to depths of ~100 m (Fig. 2). The shallow aquifer is capped by an impermeable mud layer 3–30 m thick that limits recharge (this study; [8]). The aquifer comprises a complex mud-sand stratigraphy constructed by tidal channels following the Ganges’ River progressive abandonment of the region during the late Holocene [4, 9].

### Groundwater salinity

In southwest Bangladesh groundwater specific conductivity SpC ranges from 1 to 10 millisiemens per centimeter (mS/cm, [12]), equivalent to salinities of 0.5–5.2 parts per thousand (ppt, seawater is 35 ppt). Tidal channels are an important potential source of high salinity water. Tidal channel water is a mixture of seawater from the Bay of Bengal and freshwater, with salinities up to ~15 ppt during the dry season near Polder 32 [13]. Evidence suggests that there is little or no lateral recharge from modern tidal channels. We have observed freshwater ponds adjacent to highly saline brine shrimp ponds, indicating that deltaplain mud deposits have permeabilities sufficiently low to prevent lateral exchange of water and dissolved salts. Groundwater flow modeling also suggests that the low permeability of tidal channel bank deposits and low aquifer storativity inhibits significant lateral flow of tidal channel water into the shallow aquifer underlying the polders [14].

Vertical recharge of water from saline surface sources, including brine shrimp ponds and tidal channels in the dry season, is another potential source of groundwater salinity. However, only freshwater during the wet season would be abundant enough to make recharge significant. If recharge occurs, it must be confined to small areas where the mud cap has been breached [11]. Field observations show that many larger tidal channels are scoured deeper (15–50 m) than the thickness of the surface impermeable mud layer (3–30 m) and are floored by permeable sands that may act as conduits for vertical recharge. Also, recent evidence suggests that crab burrows in surface ponds in Bangladesh may penetrate the impermeable mud cap, leading to localized freshwater recharge [15].

### Arsenic contamination of groundwater

Most of the 6–11 million tubewells in Bangladesh are sourced at 10–50 m depth in the shallow (<150 m depth) aquifer [16]. Earlier studies found that groundwater exceeded the WHO guideline of 10 μg/L As [17] in 46 % of tubewells sourced in the shallow Holocene aquifer but only 5 % in the deeper Pleistocene aquifer [16].

Arsenic dissolved in groundwater in south Asia is sourced from subsurface sediments, where it is sorbed onto Hydrous Ferric Oxides (HFOs; [18, 19]). Many previous studies have concluded that reductive dissolution of HFOs driven by microbial metabolism of organic matter results in increased concentrations of dissolved arsenic in shallow groundwaters in Bangladesh (e.g., [7]). ^14^C-rich methane and field experiments show that recent infiltration of young carbon in Bangladesh can locally drive HFO reduction and As mobilization [10, 20]. Groundwater As concentrations are highest in aquifers with low grain size and permeability that are less flushed and that are organic-rich and reducing [21].

To characterize groundwater composition and to identify the sources of salinity and arsenic, groundwater samples were collected from tubewells on Polder 32 and adjacent polders and analyzed. Arsenic values were reported to tubewell owners, who in most cases were already aware of their water quality from post-installation testing. In addition, drill core logs and radiocarbon dating of buried wood fragments were used to characterize the shallow aquifer.

## Methods

Sampling and data collection occurred throughout the study area shown in Fig. 1. Most groundwater sample locations were chosen close to populated areas, primarily around the polder’s perimeter where tubewells were located. Some groundwater samples were collected across the tidal channel on Polders 31 and 33 for comparison. Sample locations were measured with an accuracy of 50 cm using a Trimble GeoXT 6000, and well depths were provided by well owners (Table 1). Data was stored and analyzed in ESRI ArcGIS 10.2.

**Table 1:** Sample locations

Wells					Drill cores		
Well	Longitude (°)^a^	Latitude (°)	Depth (m)	Year drilled	Site	Longitude (°)	Latitude (°)
GW-10	89.433679	22.5015	22.9	2011	0	89.49508	22.56825
GW-11	89.453158	22.5454	36.6		1	89.49106	22.55319
GW-12	89.447794	22.5143		2002	3	89.492	22.51955
GW-13	89.439263	22.5254	36.6	2010	4	89.48407	22.52014
GW-14	89.495929	22.5167	48.8	2002	8	89.45905	22.47048
GW-15	89.489398	22.5353	25.9	1998	9	89.45925	22.47037
GW-16	89.482665	22.5284	19.8	2011	12	89.45217	22.43472
GW-17	89.484595	22.5328	15.2	2012	13	89.4414	22.42969
GW-19	89.481866	22.548	29	2010	14	89.43877	22.43392
GW-20	89.481554	22.5468	21.3	2011	17	89.43569	22.46502
GW-200	89.481743	22.5458			22	89.43413	22.51047
GW-21	89.483091	22.5464	22.9	2011	23	89.44209	22.50297
GW-22	89.500686	22.5336	41.8	2011	27	89.46507	22.53219
GW-23	89.500789	22.5336	38.7	2001			
GW-24	89.501026	22.5335	41.1	2011			
GW-25	89.501372	22.5335	42.1	2000			
GW-26	89.491193	22.5087	42.7	2011			
GW-27	89.49123	22.5095	45.7	2010			
GW-28	89.490784	22.5067	22.9	2010			
GW-29	89.45921	22.4704	36.6	2009			
GW-30	89.459285	22.4703	36.6	2008			
GW-31	89.45889	22.4703	44.2	2007			
GW-32	89.460374	22.4788	45.7	2011			
GW-33	89.463171	22.4591	27.4	2011			
GW-34	89.44812	22.4587	42.7	2009			
GW-35	89.448046	22.4589	42.7	2009			
GW-36	89.445487	22.4582	41.1	2011			
GW-37	89.447941	22.4364	45.7	2007			
GW-38	89.451616	22.4332	51.8	1988			
GW-39	89.451492	22.4315	42.7	2006			
GW-40	89.450372	22.4274	42.7	2005			
GW-41	89.450196	22.4278	42.7	2007			
GW-42	89.450195	22.4278	42.7	2007			
GW-50	89.472823	22.5069	33.5	2007			
GW-51	89.474373	22.5074	27.4	2011			
GW-52	89.447852	22.4362	36.6	1997			
GW-53	89.452841	22.4398	39.6	2010			
GW-60	89.491771	22.5195					

^a^Datum for latitude and longitude is WGS 1984

### Drill core collection

Thirteen cores were drilled, at locations shown in Fig. 1, using the local, hand-powered method for installing tubewells, which employs reverse-circulation flow through a 5-cm diameter PVC drill string attached to a 10-cm steel cutting shoe below surface. Locations were sited around the polder at variable spacing to assess scales of stratigraphic heterogeneity. Although no cores had identical stratigraphy, they shared the same gross architecture suggesting regional controls on their development but superimposed by local-scale dynamics. Sediment cuttings were collected over 1.5 m intervals, or 0.75 m intervals where lithology changes were noted. Core depths reached on Polder 32 ranged from 60 to 90 m below surface (Fig. 2). Mangrove wood fragments collected from drill cuttings provided 27 radiocarbon ages measured by AMS at the NOSAMS Woods Hole facility. All ages are presented in sidereal years calibrated using Calib 7.1 and the IntCal13 calibration curve (Table 2).

**Table 2:** Carbon-14 ages

Core site	Elev. (m EGM96)	NOSAMS Lab ID	Material	δ^13^C (per mil PDB)	^14^C age BP		cal yr BP	2σ upper	2σ lower
0	−30	OS-102912	Plant/wood	−28.0	7470	±30	8295	8199	8369
0	−39	OS-102913	Plant/wood	−25.0	8450	±40	9481	9422	9532
3	−8	OS-102914	Plant/wood	−28.0	150	±25	173	0	283
3	−23	OS-102915	Plant/wood	−28.0	3660	±25	3983	3901	4083
3	−62	OS-102916	Plant/wood	−28.0	8200	±35	9158	9030	9270
4	−38	OS-102917	Plant/wood	−28.4	3960	±30	4431	4296	4520
4	−54	OS-102918	Plant/wood	−29.8	9250	±40	10,425	10,275	10,554
4	−83	OS-102919	Plant/wood	−30.0	9470	±40	10,713	10,581	11,065
8	−59	OS-102920	Plant/wood	−26.8	8500	±35	9507	9470	9537
8	−84	OS-102990	Mollusc	−6.2	9670	±80	11,110	10,796	11,204
9	−3	OS-102921	Plant/wood	−27.4	240	±25	290	0	421
9	−11	OS-102975	Plant/wood	−27.1	4270	±60	4845	4728	4873
9	−22	OS-102976	Plant/wood	−29.7	7230	±70	8039	7970	8160
9	−41	OS-102977	Plant/wood	−27.6	7760	±70	8542	8448	8598
12	−27	OS-102978	Plant/wood	−27.8	7660	±70	8446	8395	8539
12	−64	OS-102979	Plant/wood	−29.1	8400	±90	9437	9303	9518
13	−14	OS-102980	Plant/wood	−26.9	2780	±80	2878	2778	2966
13	−29	OS-102981	Plant/Wood	−28.3	7590	±70	8396	8346	8432
13	−51	OS-102982	Plant/wood	−29.3	8180	±80	9121	9020	9261
17	−7	OS-102983	Plant/wood	−27.6	4340	±60	4907	4845	4972
17	−28	OS-102984	Plant/wood	−25.7	7760	±80	8538	8433	8600
17	−51	OS-102985	Plant/wood	−29.2	7950	±80	8823	8646	8983
17	−57	OS-102986	Plant/wood	−29.1	44,000	±1020	47,030	45,860	48,623
23	−22	OS-102987	Plant/wood	−27.5	7170	±70	7985	7935	8037
23	−36	OS-102988	Plant/wood	−29.9	7860	±110	8670	8541	8977
23	−54	OS-102989	Mollusc	−11.1	3340	±60	3577	3477	3677
27	−18	OS-103248	Plant/wood	−29.0	6000	±35	6840	6747	6934
27	−24	OS-103249	Plant/wood	−28.6	7360	±35	8177	8040	8309

### Water chemistry

#### Field measurements

In May 2012, October 2012 and May 2013 a portable Hydrolab 4a was used to measure physical parameters of water samples including oxidation–reduction potential Eh in millivolts (mV), pH, temperature in degrees Celsius (°C), and SpC (mS/cm). In October 2013 a portable Hydrolab DS5 was used to make the same measurements.

Platinum electrodes like those in the Hydrolab units typically only respond to a few electroactive species present at concentrations greater than ~10^−5^ molal in natural waters, usually only Fe^2+^/Fe^3+^ [22]. Thus, Eh measurements are most useful for distinguishing oxic versus anoxic conditions [37]. Comparison of Eh measurements for groundwater samples in this study with those of surface water samples [13] shows that Eh measurements made with the Hydrolab can distinguish between oxic surface waters and anoxic groundwaters.

Wells were purged at least one well volume prior to sampling. Water samples were collected by rinsing a 1 L (L) bottle, filling it, and immersing the Hydrolab Sonde for field measurements. Next, a syringe with a 0.45 μm filter was used to withdraw thirty milliliters (mL) and transfer it to a polyethylene sample bottle for Inductively Coupled Plasma (ICP) analysis. One drop of concentrated nitric acid (HNO_3_) was added as a preservative. Another 60 mL was filtered and placed in a sample bottle without preservative for ion chromatography (IC) and total organic carbon (TOC) analysis (except for samples collected in May 2012).

#### Water analysis

For all analyses an analytical blank and check standard was run every 10–20 samples and required to be within 15 % of the specified value. If the maximum concentration in the calibration standards was exceeded, then samples were diluted gravimetrically to within the targeted analytical range.

Preserved aqueous samples were analyzed for metal cation concentrations using a Varian ICP Model 720-ES ICP-OES utilizing EPA Method 6010B. Five-point standard curves were used for an analytical range between approximately 0.1 and 25 mg/L for trace metals and approximately 0.1 mg/L and 500 mg/L for major ions.

Elements below detection were reanalyzed using a Perkin Elmer Elan 6100 DRC II ICP-MS in both standard and dynamic reaction chamber (DRC) modes. Standard analysis mode was used for all analytes except for As and Se, which were run in DRC mode with 0.5 mL/min of oxygen as the reaction gas. Seven-point standard curves were used for an analytical range between approximately 0.5 µg/L and 250 µg/L and completed before each analysis.

Analyses of anions were performed on unpreserved samples using a Metrohm 881 Compact IC Pro employing ASTM Method D-4327-03. Seven-point calibration curves were generated by dilution of a multi-anion standard at 500×, 200×, 100×, 50×, 10×, 2×, and 1× and were accepted with a correlation coefficient of at least 0.995. A volume of approximately 10 mL of undiluted sample was loaded for analysis.

Analyses of organic and inorganic carbon were performed on unpreserved samples using a Shimadzu model TOC-V CPH/CPN using ASTM Method D-7573-09. Five-point calibration curves, for both dissolved inorganic carbon (DIC) and non-purgeable DOC, were generated for an analytical range between 5 ppm and 100 ppm and were accepted with a correlation coefficient of at least 0.995. A volume of approximately 20 mL of undiluted sample was loaded for analysis. DIC analysis was performed first for the analytical blank and standard and then the samples. DOC analysis was carried out separately after completion of DIC analysis. DOC analysis started with addition of 2 M hydrochloric acid to achieve a pH of 2 along with a sparge gas flow rate of 50 mL/min to purge inorganic carbon prior to analysis.

### Quality assurance/quality control

Analysis of May 2012 nitrate ${\text{NO}}_{3}^{ - }$ and DIC concentrations was compromised due to addition of HNO_3_ as a preservative (i.e., unpreserved samples were not collected in May 2012). Therefore, results for May 2012 ${\text{NO}}_{3}^{ - }$ and ${\text{HCO}}_{3}^{ - }$ concentrations are not used in the data analysis nor can charge-balance errors or saturation indices be determined for May 2012 samples.

To calculate charge balance errors ${\text{PO}}_{4}^{3 - }$ concentrations were calculated from the P concentration measured by ICP and ${\text{SO}}_{4}^{2 - }$ concentrations from S concentrations measured by ICP. Measured DIC and pH values were used to calculate concentrations of ${\text{HCO}}_{3}^{ - }$ and ${\text{CO}}_{3}^{2 - }$. For samples with complete chemical analyses (excludes May 2012 samples) the average charge-balance error was 1.2 % (Table 3).

**Table 3:** Water sample compositions

Well	Date	T (°C)	Eh (mV)	pH	SpC (mS/cm)	Al	As	B	Ba	Ca	Fe	K	Li	Mg	Mn	Mo	Na	P	S
GW-10	5/14/2012	31.9	181	6.6	5.56	0.013	0.176	0.75	0.56	80.6	1.76	41.4	0.01	78.4	0.079	0.011	1144	4.0	1.5
GW-10	10/15/2012	29.6	135	7.4	5.65		0.199	0.79	0.45	86.1	0.56	28.2	0.012	78.9	0.075	0.007	1099	3.2	0.5
GW-11	5/14/2012	28.8	165	6.9	4.59	0.004	0.062	0.76	0.11	51.9	0.66	37.1	0.007	67.1	0.064	0.003	657	5.8	1.1
GW-11	5/4/2013	28.9	99	6.9	4.18		0.027	0.97	0.10	52.4	1.00	22.8	0.168	65.3	0.061	0.007	594	5.4	15.5
GW-12	5/15/2012	33.9	179	6.6	4.95	0.022	0.196	0.52	0.57	117.0	1.02	35.2	0.01	88.6	0.070	0.003	724	1.7	1.8
GW-12	10/15/2012	28.9	122	7.3	4.95	0.028	0.167	0.60	0.55	127.4	0.82	24.9	0.012	93.5	0.071		910	1.7	0.5
GW-12	5/4/2013	27.5	−98	6.8	4.61		0.148	0.82	0.45	128.9	0.11	23.6	0.406	95.1	0.080	0.017	687	1.1	33.2
GW-12	10/24/2013	27.9	−49	6.6	5.23	0.032	0.178	0.54	0.71	110.9	3.33	24.6	0.01	89.5	0.071		682.1	2.5	5.16
GW-13	5/16/2012	31.1	135	6.5	15.23	0.049	0.020	0.56	2.56	388.2	0.75	107.3	0.016	412.8	0.299	0.002	4261	0.2	5.0
GW-14	5/17/2012	29.2	103	6.5	3.30	0.017	0.062	0.44	0.09	106.5	0.12	20.0	0.010	70.5	0.149		392	1.5	1.7
GW-15	5/17/2012	30.0	141	6.6	5.53	0.031	0.013	0.33	0.77	163.1	6.98	49.8	0.012	152.8	0.289	0.002	1251	1.5	2.4
GW-15	10/16/2012	28.2	102	7.4	5.62	0.048		0.42	0.84	184.1	6.15	37.6	0.010	158.7	0.302	0.002	856	1.3	0.5
GW-16	5/18/2012	33.4	95	6.7	6.91	0.022	0.084	0.54	0.53	138.9	0.17	15.8	0.012	46.1	0.059	0.004	1388	1.0	13.0
GW-17	5/18/2012	30.5	124	6.5	8.77	0.043	0.016	0.35	0.35	311.6	0.17	59.8	0.016	222	0.916	0.003	2134	0.1	124.9
GW-17	10/16/2012	28.4	117	7.3	8.68	0.119	0.051	0.42	0.39	326.1	12.2	41.8	0.013	212.8	0.944	0.004	1346	0.8	122.0
GW-17	5/7/2013	28.2	−85	6.6	7.80		0.040	0.7	0.34	322.1	6.12	35.9	0.534	208.3	0.907	0.022	1518	0.9	145.5
GW-17	10/25/2013	27.4	−36	6.5	8.50	0.06	0.038	0.36	0.36	265.5	14.51	41.8	0.024	198.7	0.896		1609	1.4	92.81
GW-19	5/19/2012	28.4	141	6.6	8.01	0.022	0.021	0.87	0.15	124.2	0.24	16.3	0.011	89.2	0.167		1702	6.8	3.1
GW-19	10/16/2012	28.5	130	7.3	7.99	0.030	0.031	0.84	0.27	140.7	3.87	11.4	0.013	102.4	0.235	0.002	1565	7.3	8.9
GW-19	5/6/2013	27.5	−34	6.7	7.68		0.002	1.23	0.2	136.4	0.87	8.9	0.045	94.4	0.185	0.005	1489	6.1	27.8
GW-19	10/23/2013	27.0	−37	6.5	8.22	0.042	0.004	0.85	0.24	124.9	4.47	12.2	0.013	101.9	0.23		1526	8.9	13.33
GW-20	5/19/2012	30.1	117	6.7	7.90	0.024	0.013	0.64	0.14	150.3	0.65	20.7	0.011	78.6	0.102	0.002	1753	8.7	2.6
GW-20	5/6/2013	26.9	−73	6.8	7.56		0.004	0.93	0.13	161.2	0.19	12.6	0.386	82.8	0.114	0.015	1409	6.2	35.5
GW-20 0	10/23/2013	28.1	12	7.3	29.40	0.056	0.009	2.26	0.74	192.1	0.21	38.1	0.018	382.4	0.177		7149	0.5	12.32
GW-21	5/19/2012	30.2	129	6.5	7.30	0.032	0.026	0.46	0.13	202.9	0.55	34.3	0.013	117.3	0.565	0.003	1693	3.3	65.2
GW-22	5/19/2012	29.7	183	6.6	5.57	0.021	0.080	0.61	0.76	108.7	1.11	54.6	0.010	119.8	0.084		1057	1.9	1.7
GW-23	5/19/2012	28.7	161	6.7	5.14	0.014	0.067	0.66	0.49	93	0.25	54.3	0.010	108.4	0.078		861	1.9	1.6
GW-24	5/19/2012	28.0	147	6.7	6.87	0.028	0.094	0.63	0.86	146.7	0.91	61.6	0.011	151.2	0.079	0.002	1318	1.5	2.1
GW-25	5/19/2012	28.2	164	6.7	5.26	0.011	0.053	0.69	0.3	90.1	0.35	55.8	0.009	107.9	0.059		886	2.2	1.5
GW-26	5/20/2012	32.5	324	6.9	8.89	0.034	0.030	0.57	0.58	189.7	1.35	33.9	0.013	149.4	0.16	0.002	2138	0.1	3.3
GW-27	5/20/2012	29.6	114	6.4	11.55	0.039	0.032	0.55	1.13	283.4	2.93	43.9	0.014	213.3	0.266	0.003	3009	0.6	4.3
GW-27	10/17/2012	27.9	88	7.2	11.45	0.095	0.062	0.6	1.45	291	17.48	27.5	0.011	204.9	0.274		2039	2.4	1.1
GW-28	5/20/2012	31.9	125	6.4	8.79	0.037	0.024	0.55	0.57	216	0.89	55.2	0.012	202.8	0.712		2237	0.2	3.0
GW-28	10/17/2012	28.3	88	7.3	9.23	0.094	0.017	0.62	0.78	232.2	13.33	38.2	0.009	203.1	0.621	0.003	1586	2.3	2.4
GW-29	5/21/2012	31.5	132	6.8	6.31	0.014		0.57	0.16	89.5	0.71	53.4	0.009	111.7	0.068	0.001	1342	1.2	1.2
GW-29	10/19/2012	27.3	94	7.7	6.54	0.027	0.032	0.65	0.2	103.5	2.08	39.2	0.012	123.6	0.074	0.004	1269	1.5	0.2
GW-29	5/5/2013	26.6	−84	7.1	6.21		0.001	0.95	0.2	102.6	1.81	33.4	0.476	121.8	0.073	0.018	1190	1.6	39.7
GW-30	5/21/2012	30.8	246	7.0	4.35		0.020	0.63	0.06	38.9	0.46	36.6	0.01	50.3	0.036	0.002	693	3.0	0.7
GW-30	10/19/2012	27.3	90	7.9	4.56			0.72	0.06	44.5	0.39	26.4	0.01	54.4	0.039	0.003	1002	2.9	0.4
GW-30	5/5/2013	26.6	−42	7.4	4.30		0.001	0.95	0.1	47.4	1.01	25.2	0.534	56.7	0.041	0.02	666	3.0	42.2
GW-31	5/21/2012	28.6	134	6.9	3.93		0.021	0.47	0.06	43.6	1.09	39	0.005	56.1	0.076	0.004	642	2.3	0.7
GW-31	10/19/2012	27.6	81	7.8	4.05		0.023	0.58	0.07	52.8	1.01	29.3	0.010	65.1	0.092		839	2.2	0.2
GW-31	5/5/2013	26.6	−61	7.3	3.91		0.002	0.78	0.07	54.7	1.03	27	0.368	67.3	0.097	0.018	646	2.1	32.5
GW-32	5/21/2012	28.2	143	6.5	11.74	0.040	0.115	0.62	1.44	242.5	4.8	60.2	0.016	232.5	0.678	0.004	3085	1.2	6.5
GW-32	5/6/2013	27.7	−73	6.7	14.95		0.203	0.79	2.62	392.1	3.87	42.5	0.406	344.2	0.083	0.017	3387	1.6	32.8
GW-33	5/22/2012	28.2	143	6.4	10.74	0.051	0.027	0.58	0.4	337.1	2.67	97.4	0.012	416	0.891		2835	0.6	4.3
GW-33	5/8/2013	28.1	−68	6.5	10.14		0.004	0.8	0.25	340.3	0.07	62.3	0.537	372	1.01	0.027	2136	0.8	43.3
GW-33	10/22/2013	27.5	8	6.4	11.3	0.055	0.038	0.63	0.4	324.6	10.82	72	0.028	392.4	1.218		2274	2.2	14.38
GW-34	5/22/2012	29.1	153	6.6	5.53	0.015	0.154	0.52	0.54	107.8	0.23	32.6	0.011	85.5	0.085		1005	1.9	1.5
GW-34	10/19/2012	27.7	92	7.4	5.57	0.019	0.104	0.59	0.73	113.1	3.32	21.9	0.012	86.7	0.09	0.004	1050	3.0	0.4
GW-34	5/8/2013	28.0	−77	6.7	5.24		0.107	0.89	0.62	120	2.14	21.1	0.032	92.5	0.095	0.003	925	4.1	11.5
GW-35	5/22/2012	29.3	128	6.6	7.67	0.032	0.254	0.49	0.94	176.7	0.63	36.4	0.011	136.4	0.077		1709	0.7	2.4
GW-35	10/19/2012	27.9	88	7.4	7.39	0.042	0.226	0.56	1.16	177.8	3.54	24.1	0.013	130.1	0.082		1392	1.7	0.4
GW-35	5/8/2013	29.2	−104	6.8	6.85		0.217	0.8	1	194.6	2.23	23.3	0.052	140.7	0.089	0.004	1327	2.6	34.6
GW-36	5/22/2012	29.3	149	6.7	5.67	0.022	0.114	0.49	0.56	107.2	0.72	32.3	0.011	87.5	0.065	0.002	1040	2.3	1.6
GW-36	10/19/2012	28.5	94	7.5	5.66	0.024	0.116	0.56	0.61	114.5	1.36	22.1	0.012	90.1	0.068		1110	2.3	0.4
GW-36	5/8/2013	29.5	−83	6.9	5.18		0.077	0.74	0.56	123.5	1.6	22.6	0.45	97.5	0.08	0.017	832	2.9	39
GW-37	5/22/2012	31.6	170	6.5	4.80	0.023	0.158	0.29	0.37	125.9	0.17	34.5	0.012	97.9	0.084	0.002	855	1.3	2.0
GW-37	5/5/2013	26.9	−64	6.8	4.59		0.135	0.56	0.42	145.7	2.16	25.1	0.087	110.8	0.092	0.002	748	2.8	12.1
GW-38	5/22/2012	28.1	128	6.8	5.70	0.007	0.078	0.61	0.1	65.6	0.61	40.5	0.009	77.9	0.044		1200	2.4	1.3
GW-38	5/5/2013	25.8	−58	7.1	5.54		0.057	0.87	0.14	78.1	0.83	32.2	0.211	94	0.047	0.005	989	3.0	17.1
GW-38	10/27/2013	26.3	−78	6.9	6.13	0.022	0.093	0.72	0.13	59.76	2.93	29.9	0.004	83.4	0.051		1025	3.8	3.115
GW-39	5/22/2012	28.7	118	6.6	6.75	0.034	0.104	0.3	0.62	195.2	0.44	38	0.013	131.6	0.132		1490	0.6	2.7
GW-39	10/18/2012	28.6	99	7.5	6.74	0.056	0.092	0.37	0.68	215.4	1.47	25.5	0.013	136.6	0.138		1126	0.9	0.3
GW-39	5/5/2013	25.4	−99	6.9	6.65		0.113	0.68	0.69	253.5	2.43	29.5	0.061	161.8	0.152	0.004	1279	2.7	40.9
GW-39	10/27/2013	27.2	−81	6.7	7.24	0.049	0.131	0.37	0.79	189.9	5.74	26.1	0.023	136.3	0.143		1300	2.4	8.754
GW-40	5/23/2012	29.7	149	6.6	7.04	0.041	0.048	0.39	0.72	248.5	0.91	46.2	0.012	160	0.238	0.002	1689	0.6	5.5
GW-40	10/18/2012	29.1	107	7.3	6.83	0.082	0.048	0.45	0.96	271.9	4.04	33	0.017	162.5	0.245		1036	1.5	0.3
GW-40	5/5/2013	26.1	−61	6.7	6.54		0.057	0.69	0.93	323.1	3.53	36.5	0.53	191.1	0.267	0.015	1335	2.4	42.4
GW-41	5/23/2012	29.3	134	6.6	7.94	0.046	0.030	0.25	1.4	352.7	1.39	43	0.014	186.7	0.266		1961	0.6	14
GW-41	10/18/2012	29.2	110	7.4	7.94	0.129	0.064	0.34	1.38	364.4	7.37	29.6	0.016	181	0.278	0.005	1145	1.5	7.9
GW-41	5/5/2013	26.3	−82	6.7	7.72		0.019	0.61	1.3	436.5	2.94	34.7	0.54	220.2	0.29	0.013	1582	2.3	46
GW-42	5/23/2012	29.3	134	6.6	7.94	0.048	0.043	0.26	1.43	352.4	2.2	43.7	0.013	186.9	0.269		1910	0.7	14.0
GW-50	10/17/2012	29.1	111	7.8	3.57		0.014	0.6	0.11	45.7	0.06	26.1	0.006	52	0.035	0.002	739	1.5	0.4
GW-50	5/7/2013	28.9	22	7.2	3.12		0.008	0.96	0.06	38.5	0.25	33.2	0.062	51.4	0.037	0.009	460	2.5	40.8
GW-51	10/17/2012	28.3	99	7.7	4.00		0.031	0.78	0.07	47.2	0.65	24.1	0.01	50.5	0.046	0.002	902	3.7	0.5
GW-51	5/7/2013	29.8	33	7.1	3.68		0.005	1.04	0.06	43.9	0.49	31.6	0.776	55.5	0.044	0.02	488	3.3	46.5
GW-52	10/18/2012	27.6	121	7.4	5.81	0.052	0.206	0.37	0.60	183.7	2.66	29.3	0.015	132.9	0.084	0.006	967	1.2	0.4
GW-53	10/17/2012	27.8	122	7.2	9.01	0.108	0.028	0.46	0.56	280.2	6.95	26.0	0.017	237.6	0.303		1435	0.9	0.7
GW-53	5/5/2013	25.4	−39	6.6	9.33		0.040	0.87	0.83	360.3	2.12	49.2	0.59	309.8	0.269	0.013	1916	1.4	48.6
GW-60	5/7/2013	28.9	77	7.1	4.86			0.55	0.51	192.8	0.34	36.7	7.245	147.1	0.484	0.008	940	0.9	138.2
Blank 1	10/19/2012							0.002	0.002	0.2		0.0		0.1			1		0.1
Blank 2	10/18/2012								0.002	0.007		0.004					0.3		
Blank 3	5/13/2012						0.01		0.002	0.1		0.1		0.1			1		0.7
Blank 4	5/13/2012					0.09		0.05	0.008	1.8	0.014	0.4		0.8	0.0064		14	0.01	
Minimu*m*		25.4	−104	6.4	3.1	0.004	0.001	0.25	0.057	38.5	0.058	8.9	0.004	46.1	0.035	0.001	392	0.1	0.2
Maximum		33.9	324	7.9	29.4	0.129	0.254	2.3	2.6	436.5	17.5	107.3	7.25	416.0	1.218	0.027	7149	8.9	146
Average		8.6	69	6.9	7.1	0.04	0.071	0.64	0.57	174.9	2.5	35.9	0.188	143.4	0.231	0.007	1427	2.3	19.3
Standard Deviation		1.7	97	0.4	3.5	0.028	0.065	0.27	0.5	104.4	3.4	16.6	0.815	88.6	0.271	0.007	940	1.8	31.8
Geometric Mean		28.6		6.9	6.6	0.033	0.04	0.6	0.38	143.6	1.2	32.7	0.028	122.6	0.141	0.005	1242	1.7	4.9
Method Detection Limit						0.001	0.001	0.001	0.001	0.001	0.001	0.001	0.003	0.002	0.002	0.002	0.001	0.002	0.001
WHO Guideline [17]					1.5		0.01	0.5	0.7	200	0.3	30		150	0.5		200		83
Seawater				8.1		0.001	0.002	4.5	0.01	413	6E-05	399	0.170	1290	0.0003	0.011	10,800	0.07	900

Well	Date	Si	Sr	F	Cl	Br	N0_3_	HCO_3_	DIC	DOC	Saturated minerals^a^	CIB (%)	X ^SW^ (%)						
GW-10	5/14/2012	23.8	0.7	4.5	1528								8						
GW-10	10/15/2012	21.8	0.7		1521	4.9	0.2	841	189	47.0	Hm, Ap, Gth, Dol, Cal, Qz	0	8						
GW-11	5/14/2012	26.6	0.5	4.8	898								5						
GW-11	5/4/2013	11.1	0.8	0.4	859	8.0		1165	296	34.3	Hm, Ap, Gth, Dol, Cal, Qz	−18	4						
GW-12	5/15/2012	23.2	1.0	5.1	1036	1.2							5						
GW-12	10/15/2012	22.7	1.0		1145	4.2	0.2	1028	237	49.4	Ap, Dol, Qz, Cal	2	6						
GW-12	5/4/2013	9.2	1.7	0.4	1047	8.7		1051	284	30.8	Hm, Ap, Gth, Dol, Cal, Qz	−11	5						
GW-12	10/24/2013	23.2	0.9		1049	0.8	0.2	1193	364	45.0	Ap, Hm, Mt, Gth, Qz, Dol	−16	5						
GW-13	5/16/2012	15.3	3.0	12.6	5966	11.1							31						
GW-14	5/17/2012	31.7	0.6	3.9	532								3						
GW-15	5/17/2012	20.8	1.2	5.0	1741														
GW-15	10/16/2012	19.7	1.3		1321	5.4	0.2	540	125	17.5	Hm, Ap, Gth, Dol, Cal, Qz	12	7						
GW-16	5/18/2012	23.5	0.5	6.1	2085	5.1							11						
GW-17	5/18/2012	16.0	1.5	7.3	3058	6.4							16						
GW-17	10/16/2012	14.3	1.5		2003	9.0	0.2	457	110	13.9	Hm, Ap, Gth, Dol, Cal, Qz	12	10						
GW-17	5/7/2013	2.2	2.3	0.5	2486	11.8		398	124	10.9	Hm, Dol, Gth, Cal	6	13						
GW-17	10/25/2013	17.2	1.4		2399	1.0	0.1	423	143	14.9	Ap, Hm, Mt, Gth, Qz	8	12						
GW-19	5/19/2012	32.3	0.5	6.2	2567	5.3							13						
GW-19	10/16/2012	30.2	0.6		2186	8.1	0.2	598	140	32.9	Hm, Ap, Gth, Dol, Qz, Cal	6	11						
GW-19	5/6/2013	17.5	0.6		2364	11.4	1.6	583	168	25.8	Ap, Hm, Dol, Gth, Qz, Cal	−2	12						
GW-19	10/23/2013	31.8	0.6		2326	1.0	0.4	525	175	32.6	Ap, Hm, Mt, Qz, Gth	0	12						
GW-20	5/19/2012	31.5	0.7	7.0	2592	5.6							13						
GW-20	5/6/2013	18.0	1.3		2269	11.1		675	184	28.1	Ap, Dol, Hm, Qz, Cal	−3	12						
GW-20 0	10/23/2013	10.0	4.7		10363	3.6		472	126	34.2	Hm, Mt, Gth, Dol, Qz	8	53						
GW-21	5/19/2012	28.2	0.9	6.0	2427	5.4							12						
GW-22	5/19/2012	27.0	1.0	5.0	1436								7						
GW-23	5/19/2012	29.4	0.9	5.7	1150								6						
GW-24	5/19/2012	26.1	1.3	5.9	1919								10						
GW-25	5/19/2012	28.0	0.9	5.1	1186	3.1							6						
GW-26	5/20/2012	21.9	1.1	7.3	3008								15						
GW-27	5/20/2012	22.0	1.5	9.4	4224	9.5							22						
GW-27	10/17/2012	20.0	1.4		2722	12.2	0.2	828	204	41.2	Hm, Ap, Gth, Dol, Cal, Qz	12	14						
GW-28	5/20/2012	22.5	1.5	7.4	3184	5.4							16						
GW-28	10/17/2012	20.4	1.5		2366	9.6	0.2	715	171	29.0	Hm, Ap, Gth, Dol, Cal, Qz	9	12						
GW-29	5/21/2012	24.9	0.8	6.5	1920								10						
GW-29	10/19/2012	24.2	0.9		1956	5.7	0.2	681	151	21.3	Hm, Ap, Gth, Dol, Cal, Qz	3	10						
GW-29	5/5/2013	9.8	1.7		1836	9.8		766	186	15.6	Ap, Hm, Dol, Gth, Qz, Cal	−2	9						
GW-30	5/21/2012	25.0	0.4	4.4	987								5						
GW-30	10/19/2012	25.0	0.4		1503	3.5	0.2	869	186	32.2	Hm, Ap, Gth, Dol, Cal, Qz	−6	8						
GW-30	5/5/2013	11.2	1.3		1009	8.1	1.6	1014	225	24.8	Ap, Hm, Gth, Dol, Qz, Cal	−15	5						
GW-31	5/21/2012	25.3	0.4	4.3	899	1.2							5						
GW-31	10/19/2012	25.9	0.4		1078	3.1	0.2	778	168	25.7	Hm, Ap, Gth, Dol, Qz, Cal	1	6						
GW-31	5/5/2013	12.7	1.1	0.4	937	7.9		872	197	16.8	Ap, Hm, Gth, Dol, Qz, Cal	−10	5						
GW-32	5/21/2012	20.9	2.0	10.2	4344	8.8							22						
GW-32	5/6/2013	1.7	3.7		5311	20.0		556	169	16.4		8	27						
GW-33	5/22/2012	33.1	2.8	9.9	4034	8.8							21						
GW-33	5/8/2013	17.6	3.2		3431	13.5		590	202	16.2	Dol, Qz, Cal	10	18						
GW-33	10/22/2013	32.6	0.3		3447	1.3	0.1		236	25.4	Ap, Hm, Mt, Gth, Qz	12	18						
GW-34	5/22/2012	25.1	0.8	5.9	1423	2.8							7						
GW-34	10/19/2012	23.3	0.8		1553	4.7	0.2	928	210	40.7	Hm, Ap, Gth, Dol, Qz, Cal	−2	8						
GW-34	5/8/2013	11.5	0.8		1348	9.0		874	250	23.2	Ap. Hm, Dol, Gth, Qz, Cal	−5	7						
GW-35	5/22/2012	20.0	1.3	7.9	2419	6.5							12						
GW-35	10/19/2012	19.4	1.3		2119	6.9	0.2	891	206	31.4	Hm, Ap, Gth, Dol, Cal, Qz	2	11						
GW-35	5/8/2013	8.0	1.4		2020	10.6		899	245	18.4	Hm, Dol, Gth, Cal, Qz	0	10						
GW-36	5/22/2012	21.5	0.8	5.7	1403	0.8							7						
GW-36	10/19/2012	19.6	0.8		1435	4.9	0.2	1009	226	44.9	Hm, Ap, Gth, Dol, Cal, Qz	2	7						
GW-36	5/8/2013	7.7	1.4		1319	8.9		1055	271	28.3	Ap, Hm, Dol, Gth, Cal, Qz	−10	7						
GW-37	5/22/2012	30.5	1.0	5.1	1202	1.1							6						
GW-37	5/5/2013	16.7	1.3		1165	9.1		782	212	27.4	Ap, Hm, Dol, Gth, Qz, Cal	−2	6						
GW-38	5/22/2012	29.0	0.7	6.0	1617	2.5							8						
GW-38	5/5/2013	16.8	1.1		1492	9.5		826	198	24.0	Ap, Hm, Dol, Gth, Qz, Cal	−4	8						
GW-38	10/27/2013	29.5	0.7		1480	0.8	0.1	939	242	35.6	Ap, Hm, Mt, Gth, Qz, Dol	−6	8						
GW-39	5/22/2012	21.7	1.4	6.2	2116	3.7							11						
GW-39	10/18/2012	20.2	1.5		1709	5.8	0.2	673	154	22.8	Hm, Ap, Gth, Dol, Cal, Qz	8	9						
GW-39	5/5/2013	9.4	1.7		1938	9.8		679	180	15.7	Hm, Dol, Gth, Qz, Cal	7	10						
GW-39	10/27/2013	21.3	1.5		1936	0.9	0.1	746	217	26.4	Ap, Hm, Mt, Gth, Qz, Dol	3	10						
GW-40	5/23/2012	31.4	1.8	6.3	2249	4.5							12						
GW-40	10/18/2012	30.2	1.9		1509	6.0	0.2	645	153	22.0	Hm, Ap, Gth, Dol, Cal, Qz	14	8						
GW-40	5/5/2013	18.6	2.8		1980	9.9	1.5	585	172	15.2	Hm, Dol, Gth, Qz, Cal	11	10						
GW-41	5/23/2012	28.7	2.1	7.5	2757	4.9							14						
GW-41	10/18/2012	27.5	2.1		1506	7.3	0.2	659	157	22.4	Hm, Ap, Gth, Dol, Cal, Qz	20	8						
GW-41	5/5/2013	15.9	3.1		2410	10.9		578	172	15.0	Hm, Dol, Qz, Gth, Cal	12	12						
GW-42	5/23/2012	29.0	2.2	6.6	2728								14						
GW-50	10/17/2012	24.3	0.4		1106	3.0	0.2	774	166	35.4	Ap, Hm, Gth, Dol, Qz, Cal	−7	6						
GW-50	5/7/2013	15.2	0.5		686	7.5		881	201	23.1	Ap, Hm, Gth, Dol, Qz, Cal	−18	4						
GW-51	10/17/2012	31.8	0.4		1339	2.9	0.2	1003	217	43.3	Hm, Ap, Gth, Dol, Qz, Cal	−9	7						
GW-51	5/7/2013	20.6	1.2		746	7.6		1116	262	25.8	Ap, Hm, Gth, Dol, Qz, Cal	−23	4						
GW-52	10/18/2012	27.6	1.4		1281	5.8	0.2	826	190	41.3	Hm, Ap, Gth, Dol, Cal, Qz	9	7						
GW-53	10/17/2012	27.3	1.4		2101	8.5	0.2	739	182	29.2	Hm, Ap, Gth, Dol, Qz, Cal	13	11						
GW-53	5/5/2013	17.5	2.7	0.4	3017	12.4		641	203	21.1	Hm, Dol, Qz, Gth, Cal	10	15						
GW-60	5/7/2013		1.1	0.4	1425	9.3		393	95	10.9	Hm, Gth, Dol, Cal	6	7						
Blank 1	10/19/2012				1		0.17		0.5	2.1									
Blank 2	10/18/2012				0.4				23.8	4.9									
Blank 3	5/13/2012			0.32	1				0.21	4.9									
Blank 4	5/13/2012	1.6	0.01	1.97	10				0.07	4.5									
Minimum		1.7	0.3	0.4	532	0.8	0.1	393	95	10.9									
Maximum		33.1	4.7	12.6	10363	20	1.6	1193	364	49.4									
Average		21.6	1.3	5.5	2076	6.6	0.3	766	195	27		1.2	11						
Standard deviation		7.3	0.8	2.8	1368	3.8	0.4	206	50	10.1		10	7						
geometric mean		19.7	1.1	4.0	1803	5.2	0.2	737	188	25.2									
Method detection limit		0.003	0.004	0.007	0.001	0.001	0.001		0.007	0.018									
WHO guideline [17]					250		50	384	0.007	0.018									
Seawater		2.8	7.6	1.3	19500	67.0		142		0.1									

All concentrations in mg/L

HCO_3_− and saturation indices calculated using GWB v. 9. X^SW^ (%) is the estimated percentage of seawater in the groundwater mixture (see text)

*SpC* specific conductivity, seawater composition from [32] except for HCO_3_− and DOC concentrations from [33]

*Hm* hematite; *Ap* apatite; *Gth* goethite; *Dol* dolomite; *Cal* calcite; *Qz* quartz; *Mag* magnetite; *DIC* dissolved inorganic carbon; *DOC* dissolved organic carbon

^a^CIB (%) = charge imbalance error. Saturated minerals have saturation index (= log IAP/Ksp) >0

Method detection limits are given for each analyte in Table 3. Sample blanks consisting of deionized water were collected in-field during each field campaign and analyzed, yielding concentrations that were consistently lower than in samples (Table 3). For the three elements that were analyzed by both methods (B, As and Mn) ICP-OES and ICP-MS analysis results showed excellent agreement, so only ICP-MS results are given in Table 3. For example, the average % difference was 5 % for B. The average % difference for As was higher at 18 % because many samples were near the method detection limit for ICP-OES.

## Results

### Subsurface stratigraphy

**Fig. 3:**
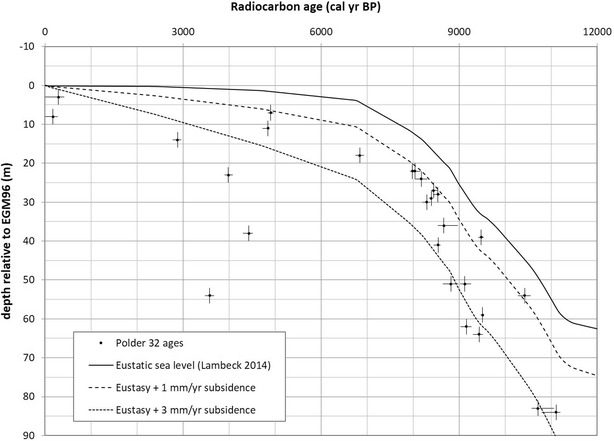
^14^C ages measured from mangrove wood fragments from Polder 32 plotted as a function of depth in meters compared to the eustatic sea level curve of [38] with 0, 1, and 3 mm/year of subsidence. Elevations are normalized to EGM96 datum. Results show rapid aggradation and aquifer construction during early Holocene sea-level rise, with slower aggradation and sea-level rise since the mid-Holocene. The several deep (23–54 m) radiocarbon ages around 4000 ± 500 YBP correspond with a local channel scour

Drill cores from thirteen locations (Fig. 1) reveal a stratigraphy dominated by two principal facies (Fig. 2): (a) a relatively coarse lower unit comprising up to 60 m of sand-dominated lithology with scattered thin (<2 m) mud layers; and (b) an overall finer upper unit comprising 30–40 m of alternating 5–20 m thick sand and mud deposits. The lower unit is characterized by thick, clean sands up to medium grain size (250–500 µm). The presence of medium sand, low sediment Sr concentration (80–110 ppm), and the thick, open sandy architecture indicate that these sediments were deposited by the main Ganges River channel [9]. The abundant wood fragments and intertidal gastropods (*Littorina* sp.) further constrain this setting to the lower, tide-influenced reach of the paleo-rivermouth. Radiocarbon ages indicate the timing of deposition to extend from ~11,000 calendar years Before Present (YBP) at the base of the cores (~90 m depth) to ~8500 YBP near the top of the lower unit at 30–40 m depth (Table 2). These results reflect mean sediment accretion rates of ~2 cm/yr during the early Holocene, which were sufficient to keep pace with rapid post-glacial sea-level rise (Fig. 3). The age-elevation distributions from this time also plot below eustatic sea level and suggest local subsidence or compaction rates of no more than 1–3 mm/yr (Fig. 3). Most tubewells in the area are screened in the upper part of this early Holocene aquifer, in sediments deposited 8000–9000 YBP, although ^14^C ages of the associated groundwaters are considerably younger at 1500–5000 YBP [11].

Beginning ~8500 years ago at 30–40 m depth there is a major change in stratigraphy that is characterized by the appearance of alternating thick layers of muds and sands in the upper unit. The increase in mud within the stratigraphy indicates that the main river mouth had avulsed or progressively migrated to another portion of the delta and by ~6500 YBP no longer occupied this location. Subsequent deposition of thick mud layers with abundant wood fragments reflect a mangrove-forested, intertidal delta plain, like that found today in the adjacent Sundarbans. The fine sand deposits within the same unit represent the high-energy tidal channels that interlace the delta plain. Most of the tubewells on Polder 32 are screened near the base of this heterolithic upper unit in sediments deposited <7000–8000 YBP. Mineralogy of these deposits are typical for the region, dominated by quartz and feldspars with variable contributions of carbonate, amphibole, garnet, epidote, biotite, and muscovite in the sand fraction and illite, smectite, kaolinite, chlorite in the silty muds [1, 39, 43, 44].

### Water compositions

All sampled tubewells (Fig. 1) are screened at depths of 15–52 m (Table 1; Fig. 2), meaning that all of our groundwater samples are from the shallow aquifer that is the most commonly utilized groundwater resource in this region. Samples collected in May are taken to represent the dry season and October the wet season.

Measured concentrations of most elements displayed a lognormal distribution. This was confirmed by transforming the concentrations to their base 10 logarithms and testing for normality using Kolmogorov–Smirnov tests. All statistical tests and plots therefore use log_10_ values of concentrations, and cutoffs for statistical tests are at a significance level P = 0.05, meaning that any differences referred to in the following discussion are significant at the 95 % level, and errors are stated as 95 % confidence limits.

**Fig. 4:**
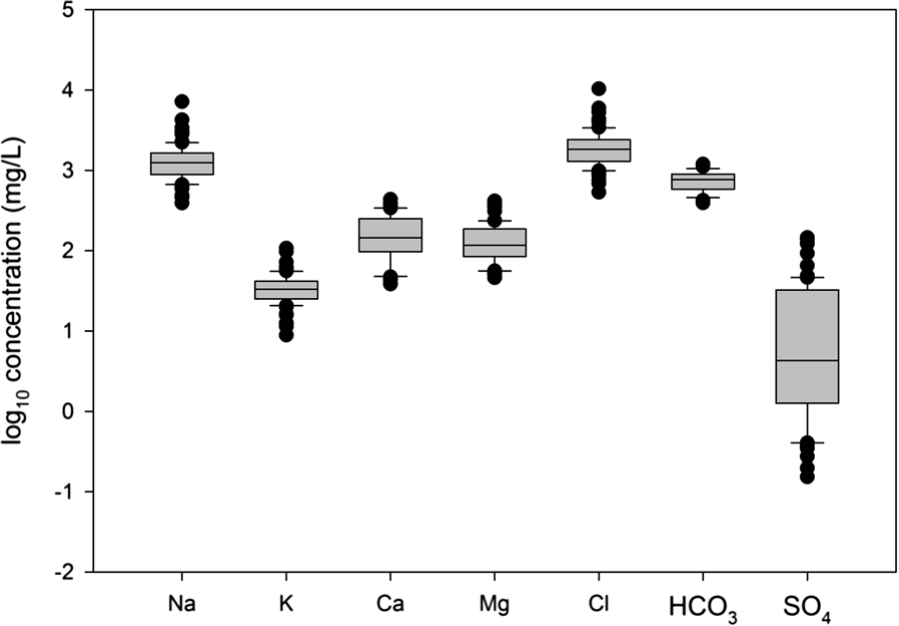
*Boxplot* showing distribution of concentrations of major cations and anions in groundwater samples. Concentrations of ${\text{HCO}}_{3}^{ - }$ calculated from measured DIC and pH using The Geochemists Workbench v. 9

All groundwater samples are Na–Cl type and have near neutral pH, with values ranging from 6.4 to 7.9 with an average value of 6.9 (Table 3). Specific conductivity SpC, which we use as a measure of salinity, ranges from 3.1 to 29.4 mS/cm with an average value of 7.0 mS/cm. Average concentrations of cations in groundwater occur in the order Na^+^ > Ca^2+^ > Mg^2+^ > K^+^ and anions Cl^−^ > ${\text{HCO}}_{3}^{ - }$ > ${\text{SO}}_{4}^{2 - }$ (Fig. 4). Mineral saturation indices were calculated using the Spec8 program in the Geochemists Workbench v. 9. Most groundwater samples are saturated to oversaturated in hydroxyapatite, the HFO goethite (FeO(OH)), dolomite, calcite and quartz, with all but dolomite known to be present in the shallow aquifer sediments (Table 3).

### Conservative elements

**Fig. 5:**
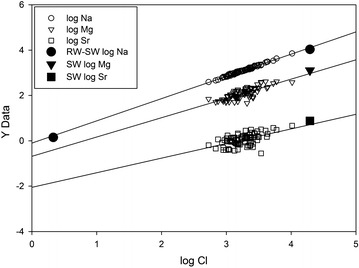
Concentration of Cl plotted versus concentrations of conservative elements Na, Mg, and Sr with best-fit linear regression lines. The large *filled symbols* at high Cl concentration represent seawater [32], while the *filled circle* at low Cl concentration represents the average Na concentration in two collected rainwater samples [13]. All concentrations are log_10_ values in mg/L

**Fig. 6:**
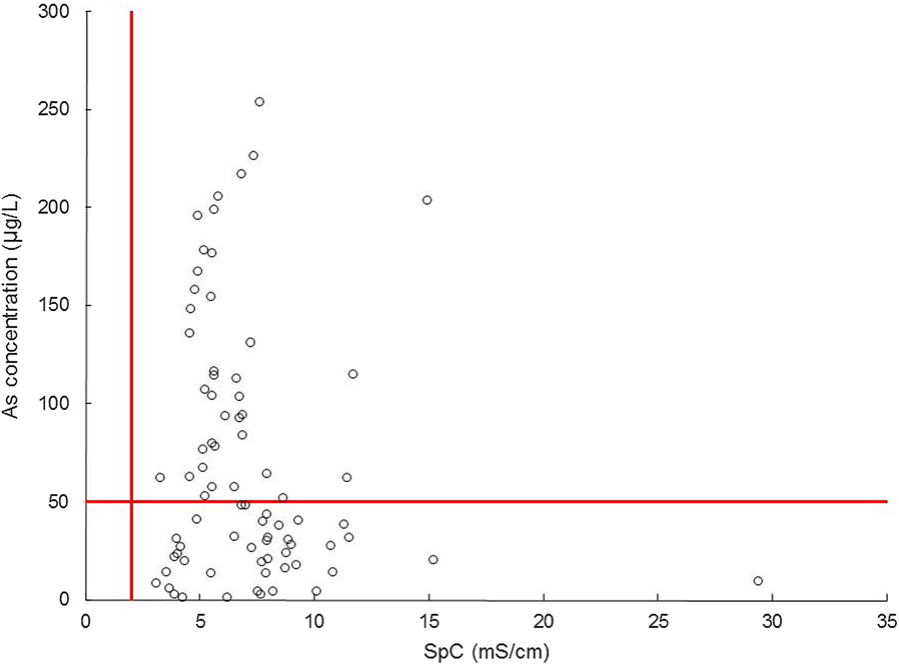
Specific conductivity SpC in mS/cm plotted versus As concentration in μg/L for groundwater samples from tubewells. Compared to Bangladesh government guidelines shown as red lines, 100 % of groundwater samples exceed the salinity guideline of 2 mS/cm, while 46 % exceed the As guideline of 50 μg/L

Linear correlations between the concentrations of Na, Mg, and Sr with Cl indicates that these elements behave conservatively in groundwater (Fig. 5). Linear regression lines for each cation-Cl pair appear to represent mixing lines. Seawater falls close to the mixing lines for each cation-Cl pair, suggesting that seawater is a mixing endmember. Other lines of evidence support seawater being the saline endmember. The average Cl (mg/L)/B (mg/L) of 273 ± 66 (95 % CL) in our tubewell samples is similar to that of 290 for seawater. Also, at a given Cl concentration the Cl/Br of our groundwater samples is much lower than the mixing line for West Bengal Salt obtained from evaporite beds in the region, but similar to the seawater mixing line [24], suggesting that evaporites are not the source of dissolved salts. Finally, tidal channel water that deposits most sediments in the region is a mixture of seawater, and freshwater from river discharge and local runoff, and is likely trapped as pore water during sediment deposition [13]. The dilute mixing end member could be rainwater, as the average concentration in two rainwater samples collected in the field [13] plots right on the mixing line for Na–Cl (Fig. 5), but it could be tidal channel water, as tidal channel water samples also fall on the mixing line [13]. The Bangladesh government guideline for salinity of 2 mS/cm SpC [26] is exceeded by 100 % of our groundwater samples (Fig. 6).

### Nonconservative (redox-sensitive) elements

In contrast to salts, many redox-sensitive elements behave non-conservatively, meaning their proportions are highly variable. Of greatest concern is the high concentration of toxic arsenic in groundwater samples. Arsenic concentrations range from 1 to 254 μg/L with a geometric mean of 40 μg/L (Table 3). Of the groundwater samples analyzed, 83 % exceed the WHO guideline of 10 μg/L As and 46 % exceed the Bangladesh government guideline of 50 μg/L [27] (Fig. 6). In contrast, the geometric mean Mn concentration of 141 μg/L is lower than the WHO guideline of 500 μg/L. The mean DOC concentration is 27 ± 3 mg/L, which is unusually high for groundwater [28] and much higher than for all other water types we have analyzed using the same methods [13], including field blanks that yielded an average concentration of 4 ± 2 mg/L (Table 3). Generally poor correlations between Eh, concentrations of reducing agents (DOC), and concentrations of metals with variable oxidation states (As, Fe, Mn and S) suggests that redox disequilibrium is the norm.

## Discussion

### Groundwater flow

Since the hydraulic head gradients in southern Bangladesh are very low, groundwater flow velocities are low [10]. Heavy rainfall and high river discharge during the monsoon replenishes groundwater aquifers through both vertical and lateral recharge, but in the coastal zone recharge is likely limited by the impermeable surface mud layer. Within the shallow aquifer sand beds are not laterally continuous (Fig. 2), and interbedded silt layers cause groundwater flow to slow and change direction, causing groundwater flow rates to be low. The low flow rate and variable permeability inhibit mixing, allowing the persistence of spatial variability in groundwater composition.

The variable thickness of the mud cap observed in the sediment cores (Fig. 2) is consistent with results from inversion of electromagnetic survey measurements, which show that in some areas of Polder 32 the mud cap is thin or non-existent [11]. Areas where the surface mud layer pinches out are likely sites for localized recharge of the shallow aquifer with rainwater or surface water, which would result in formation of freshwater lenses floating on the denser brackish groundwater. Inland streams (natural streams and irrigation ditches) and tidal channels are points of low elevation, so they are the most likely entry points for infiltrating surface water. These local depressions are usually dry in May but are filled with freshwater in the wet season [13]. Thus, fresh water is more likely to infiltrate into the subsurface than brackish water.

### Groundwater composition

#### Spatial variation

**Fig. 7:**
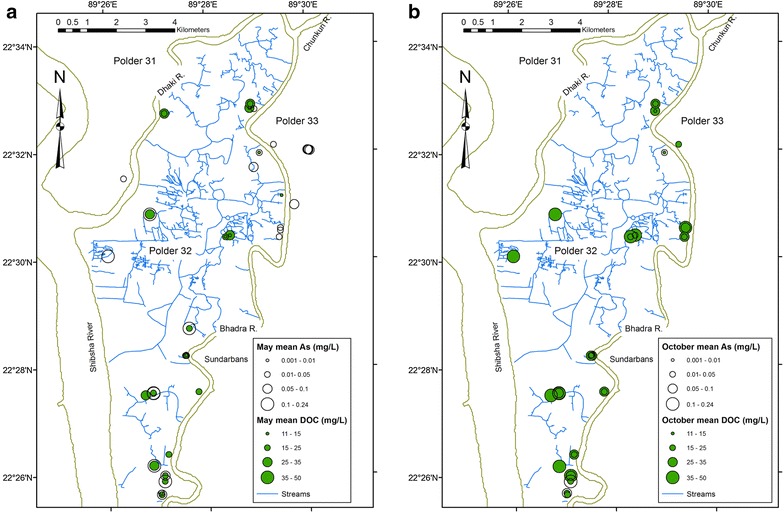
Maps of average measured As and DOC concentrations (mg/L) of groundwater samples collected from each tubewell in 2012-13. **a** May. **b** October

The subsurface stratigraphy beneath Polder 32 shows a high degree of spatial heterogeneity (Fig. 2). Likewise, groundwater composition shows high spatial variability in specific conductivity (Fig. 1) and concentrations of As and DOC (Fig. 7). For example, one tubewell may yield saline water, while a tubewell next to it drilled to roughly the same depth yields fresh water. Such local-scale heterogeneity may not be unexpected for non-conservative, redox-sensitive elements such as As (e.g., [16, 18, 45]), but it is perhaps more surprising for conservative elements in a Holocene-age aquifer. This suggests that the spatial distributions of salinity and As concentrations cannot be distinguished from a random distribution. To test this, we used the Geostatistical Analyst extension in ESRI ArcMap 10.2 for construction of spatial trend plots and semivariograms for select compositional variables. Semivariograms plot the semivariance, which is proportional to 1-autocorrelation, as a function of distance, in this case between every possible paired combination of tubewells. Semivariograms for all tested compositional variables show a random pattern, suggesting that there is no spatial autocorrelation of element concentrations in groundwater and that the spatial variation is just noise. This is supported by the value of nugget being close to one, which suggests that compositional variability occurs on a smaller spatial scale than the sampling distance [30, 31]. Spatial interpolation assumes that samples that are spatially close have similar values, i.e., there is regional dependence, which is the same as spatial autocorrelation at low lags [45]. Because our data show no spatial autocorrelation, spatial interpolation is not warranted, so we cannot construct meaningful contour maps of groundwater composition. Similar conclusions were drawn by The Bangladesh National Hydrochemical Survey of 1998–1999 for comparable spatial scales [16].

There are other obstacles to making accurate spatial generalizations about groundwater composition. One problem is that groundwater composition in the polder interior is poorly constrained due to a lack of tubewells (Fig. 1). Another problem is that the wells were drilled to different depths, so tubewell water samples were recovered from different depths. Interpolating As concentrations from all wells to create a 2D surface would require the assumption that there is no vertical heterogeneity, which is false (Fig. 2).

**Fig. 8:**
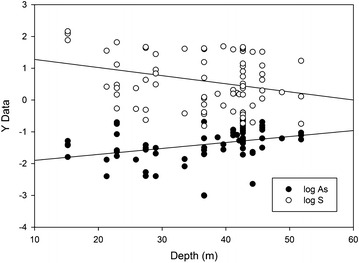
Screened depths of groundwater wells versus log_10_ concentrations of As and S in mg/L

While tubewell salinity and As concentrations show no coherent surface trends, some element concentrations change systematically with depth. Statistically significant correlations with increasing depth include an increase in As and decrease in S (Fig. 8). These correlations were significant even when making corrections for multiple comparisons by performing bootstrap calculations in SPSS v. 23: the bias-corrected 95 % confidence intervals for the Pearson correlation coefficient for log_10_As were 0.18–0.59 and for log_10_S −0.04 to −0.60. No consistent trend with depth is observed for salinity (SpC), Eh or pH.

### Source of salts

Salinity of groundwater from tubewells on and near Polder 32 is spatially variable (Fig. 1). The average salinity of groundwater in the area of Polder 32 is ~10 ppt, roughly 1/3 that of seawater and similar to the salinity of tidal channel water in the dry season. We suggest that groundwater in this region is saline because it comprises connate tidal-channel water deposited with the sediments during Holocene aquifer development, similar to what is observed today. The concentrations of components of soluble salts such as the alkali element Na, alkaline earths Mg and Sr and halogen Cl are all highly correlated, indicating that they behave conservatively. Groundwater samples define linear arrays on bivariate plots of these elements that most likely represent mixing between saline and dilute endmembers (Fig. 5).

**Fig. 9:**
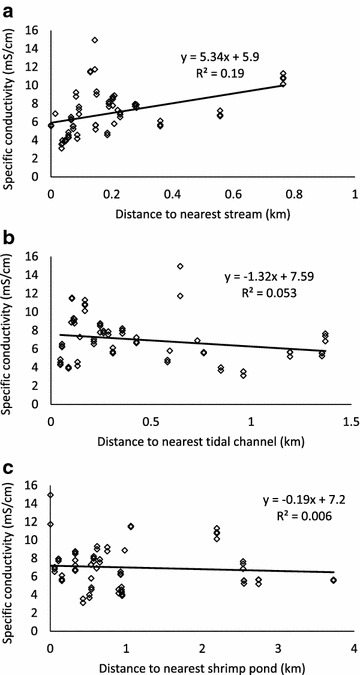
Specific conductivity of groundwater in mS/cm as a function of shortest distance in km to (**a**) a stream. **b** a tidal channel. **c** a shrimp pond

Although semivariograms and trend analysis suggest that groundwater salinity shows no spatial autocorrelation, it is still possible that salinity may show a systematic dependence on distance from freshwater or brackish water sources, allowing identification of sources. Potential sources include streams within polder embankments, tidal channels that surround the polders, and brine shrimp ponds. A proximity analysis performed in ArcGIS 10.2 shows that although correlation coefficients are low and not significant, the correlation between groundwater SpC and distance to the nearest stream is strongest and positive, suggesting that streams may be sources of freshwater recharge to groundwater (Fig. 9a). No correlation was observed between groundwater SpC and freshwater pond distance. Conversely, we observe weak negative correlations between SpC and distances to tidal channel (Fig. 9b) and shrimp ponds (Fig. 9c), suggesting that the latter are potential brackish water sources. It is possible that some brackish water intrusion from the tidal channels and shrimp ponds into the shallow aquifer has occurred. However, the bulk of the evidence suggests that dissolved salts in groundwater were inherited from paleo-tidal-channel water at the time of sediment deposition, with salinity variations due to spatially variable amounts of freshwater recharge resulting from variations in thickness of the impermeable surface mud layer. This interpretation is consistent with modeling of Holocene groundwater evolution around Polder 32, which demonstrates that slow but spatially variable recharge can account for observed groundwater age and salinity patterns[11].

Together, highly variable flow paths and localized recharge result in groundwater compositions that vary greatly spatially (Figs. 1, 7 and 9) and with depth (Fig. 8). Measured groundwater radiocarbon ages from [11] are also spatially variable (Fig. 2). These ages are younger than the depositional (radiocarbon) ages of their host sediments, suggesting mixing of old, connate brackish tidal channel water and younger meteoric water. Groundwater recharge by (contaminated) surface water is consistent with reports of contamination of tubewell water by pathogens on Polder 32 and with previous studies that showed that surface ponds in the region are a source of recharge water and labile carbon [20]. Assuming the initial concentration of ^14^C has not changed over time (not strictly true), the age of a groundwater t_gw_ that is a connate water—modern freshwater mixture is related to the weight fraction of connate water X^CW^ by the equation:

1 $$t_{gw} = \frac{{ln\left( {X^{CW} e^{{ - \lambda t_{Cw} }} + (1 - X^{CW} )} \right)}}{ - \lambda }$$

Adjusting X^CW^ until t_gw_ equals the average ^14^C groundwater age of 2921 Y [11] yields an average weight fraction of connate water of 0.46 or 46 wt%. The four measured ^14^C groundwater ages yield a range of 19–77 wt% connate water.

This range of proportions overlaps with the range of Na and Cl concentrations in samples plotted in Fig. 5, with samples closer to the seawater endmember having higher proportions of seawater that was a component of the connate water. If groundwater is assumed to be a binary mixture of seawater and rainwater, then for conservative elements such as Cl a mass balance equation can be used to estimate the proportion of seawater in groundwater X^sw^:

2 $${\text{C}}_{\text{Cl}}^{\text{gw}} = {\text{ X}}^{\text{sw}} {\text{C}}_{\text{Cl}}^{\text{sw}} + \, \left( { 1- {\text{X}}^{\text{sw}} } \right){\text{C}}_{\text{Cl}}^{\text{rw}}$$

where C_Cl_^gw^ and C_Cl_^rw^ are the average concentrations of Cl in groundwater and rainwater, and X^sw^ is the weight fraction of seawater. Setting C_Cl_^sw^ = 19,500 mg/L [32], average C_Cl_^gw^ = 2076 mg/L (Table 3) and C_Cl_^rw^ = 2.14 mg/L [13] gives X^sw^ = 0.11, which suggests a lower average proportion of seawater of 11 %. Individual analyses yield a range of 3 to 53 % seawater (Table 3). Note that while seawater is the ultimate source of dissolved salts, the water trapped during sediment deposition was a brackish tidal water. Variable amounts of modern freshwater recharge further diluted the groundwater.

Although no firm conclusions can be drawn on the basis of only four samples, the ages measured by Worland et al. [11] do not correlate with our groundwater salinities measured at the same tubewells. However, tritium contents show a negative correlation with specific conductivity, consistent with the idea that samples with the greatest amount of recharge by modern freshwater (highest tritium contents) have been the most diluted. The presence of modern recharge implies that the shallow groundwater aquifer is susceptible to contamination by surface waters, most likely in those areas where the impermeable clay cap pinches out. Also, if the extraction rate for freshwater wells exceeds the recharge rate, then freshwater lenses in the subsurface may shrink until tubewell water becomes saline. Unfortunately, the recharge rate is not known, so we cannot estimate sustainable pumping rates. However, extraction rates are certainly low on Polder 32, as there are no large-volume irrigation wells and most hand-pumped wells were only installed after 2009 Cyclone Aila and receiving limited use due to the generally saline groundwater [46].

### Source of As and cause of As mobility

**Fig. 10:**
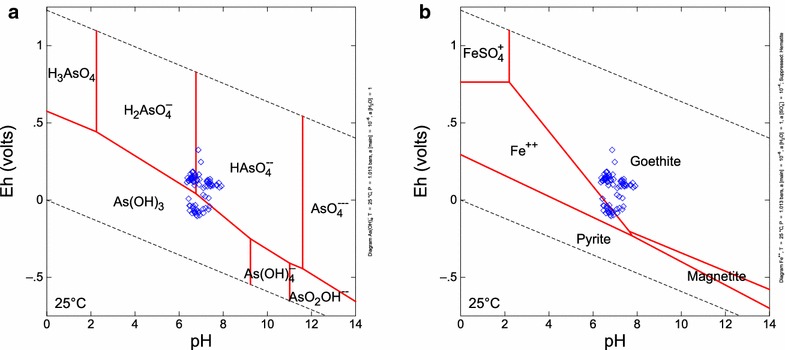
Eh-pH diagrams calculated in the Geochemists Workbench v. 9 Act2 program using the default thermodynamic database thermo.dat with temperature = 25 °C, pressure = 1.013 *bars*, and activity of H_2_O = 1.0. **a** As speciation. Activity of As species 10^−6^. **b** Fe and S species, hematite suppressed, activity of Fe^2+^ = 10^−6^, and activity of SO_4_^2−^ = 10^−4^

Most of our tubewell samples had As concentrations higher than the WHO guideline of 10 μg/L, although many remain below the Bangladesh guideline of 50 μg/L. The oxidation state of dissolved As and stoichiometry of As-compounds can be inferred from measured Eh and pH measurements, thermodynamic data, and the assumption of chemical equilibrium. Uncertainty arises from disequilibrium between As(III), As (V) and other redox species [40] and the poor response of Pt electrodes to many aqueous redox couples [22, 37]. An Eh-pH diagram for As shows that at equilibrium the dominant As species in the most reduced groundwater samples is the neutral complex As(OH)_3_ with As having a +III valence (Fig. 10a). In more oxidized groundwater samples As is in the +V valence state and the dominant species are ${\text{HAsO}}_{4}^{ - 2}$ or ${\text{H}}_{ 2} {\text{AsO}}_{4}^{ - }$. The relative proportions of As(III) and As(V) affect As toxicity and mobility. As(III) is more toxic than As(V) [41]. The mobility of the charged As(V) complexes would be reduced by adsorption to a greater extent than for the uncharged As(III) complex As(OH)_3_, although adsorption of As(V) becomes less important at pH values above 7 ([42]; the average pH of our groundwater samples is 6.9). Thus, As should be most toxic and mobile in the most reduced (lowest Eh) groundwaters generally found in grey, reducing Holocene sediments.

Groundwater samples do not plot in the pyrite stability field (Fig. 10b) and so are not saturated in pyrite, which suggests that pyrite is not widely present in the aquifer. This is consistent with mineral saturation indices that show all groundwater samples are undersaturated in pyrite (Table 3). Most groundwater samples plot in the stability field of goethite, an HFO mineral (Fig. 10b), consistent with calculated saturation indices showing most groundwater samples are oversaturated in goethite (Table 3). Many groundwater samples plot near the boundary between goethite and aqueous ferrous iron, suggesting that reductive dissolution of HFOs and resulting mobilization of adsorbed As is likely.

Mixing calculations can be used to estimate the theoretical concentration of redox species in the initial seawater-rainwater mixture (i.e., in trapped tidal channel water). Estimates of X^SW^ (Table 3) and sulfate concentrations ($C_{S{O_4}}^{SW} = 2700\,mg/L$ [32] and $C_{S{O_4}}^{rw} = 1.5\,mg/L$ [13]) were substituted in Eq. (2) to calculate the theoretical concentrations expected if sulfate behaved conservatively. In 76 samples the actual sulfate concentration was less than the theoretical, implying that sulfate reduction removed sulfate from the mixture; according to Buschmann and Berg [23] these are classified as sulfate-reducing. The five samples for which the actual sulfate concentration was greater than the theoretical were classified as iron-reducing because for all five samples Fe > 0.2 mg/L (Table 3). Groundwater As concentrations would likely be higher than observed if conditions were more reducing, i.e., in the methanogenic zone rather than the sulfate-reducing and iron-reducing zones [23].

Previous work in this region has shown that As concentrations in aquifer sediments are highly correlated with Fe content [16] because As in sediments is primarily sorbed to HFOs [18] and immobile, and that biologically active organic carbon can reduce HFOs and mobilize As [20]. HFOs are present in Holocene sediments in the adjacent Sundarbans [18] and throughout the region [7]. Furthermore, DOC concentration in groundwater samples is higher than in any surface water types in the vicinity ([13, 25]). Our measured DOC concentrations ranged from 11 to 49 mg/L, which is highly comparable with average porewater DOC of 19–36 mg/L measured in four different sediment types in the Sundarbans [18]. This similarity is also consistent with our hypothesis that modern shallow groundwater in Polder 32 began as DOC-rich connate porewater trapped in HFO-bearing surface sediments at the time of deposition, as is occurring in the modern Sundarbans. Reactive organic carbon can be preserved in permanent wetlands that become anoxic, and subsequent microbial oxidation leads to reduction of arsenic-bearing HFOs and As release according to the reaction [34]:

3 $${\text{CH}}_{ 2} {\text{O }} + {\text{ 4FeOOH}} - \left( {{\text{H}}_{ 2} {\text{AsO}}_{ 4} } \right)_{\text{x}} + \, \left( { 7 { } + {\text{ 3x}}} \right){\text{H}}^{ + } = {\text{ 4Fe}}^{ 2+ } + {\text{ HCO}}_{ 3}^{ - } + \, \left( { 6 { } + {\text{ x}}} \right){\text{H}}_{ 2} {\text{O }} + {\text{ xH}}_{ 3} {\text{AsO}}_{ 3}$$

In Eq. (3) CH_2_O represents DOC. Equation (3) explains the following observations:

1. Groundwater iron and arsenic concentrations are higher than expected for a seawater-rainwater mixture (seawater concentrations given in Table 3, rainwater concentrations are negligible).
2. Groundwater bicarbonate concentrations are also higher than expected for a seawater-rainwater mixture (the geometric mean concentration of ${\text{HCO}}_{3}^{ - }$ in groundwater of 737 mg/L being higher than the seawater concentration of 142 mg/L [33]), although other reactions such as carbonate dissolution/precipitation may increase the concentration of ${\text{HCO}}_{3}^{ - }$.

While Na/Cl and Cl/Br ratios indicate that the groundwater has a paleo-seawater component, the geometric mean S concentration of 5 mg/L is much lower than the mean seawater concentration of 900 mg/L [32]. However, sulfide loss was not caused by sulfide precipitation, as mineral saturation calculations indicate that groundwater samples are not saturated in sulfide minerals such as pyrite (Table 3). Sulfur therefore may have been lost by reduction of sulfate to H_2_S and then loss of H_2_S in the aquifer or in the tubewells before or during sampling:

4 $${\text{SO}}_{ 4}^{ 2- } + {\text{ 2CH}}_{ 2} {\text{O }} = {\text{ H}}_{ 2} {\text{S }} + {\text{ 2 HCO}}_{ 3}^{ - }$$

Combining Eqs. (1) and (2) we obtain:

5 $$\begin{aligned} 3 {\text{CH}}_{ 2} {\text{O }} + {\text{ 4FeOOH}} - \left( {{\text{H}}_{ 2} {\text{AsO}}_{ 4} } \right)_{\text{x}} + {\text{ SO}}_{ 4}^{ 2- } + \, \left( { 7 { } + {\text{ 3x}}} \right){\text{H}}^{ + } \hfill \\ = {\text{ 4Fe}}^{ 2+ } + {\text{ H}}_{ 2} {\text{S }} + {\text{ 3HCO}}_{ 3}^{ - } + \, \left( { 6 { } + {\text{ x}}} \right){\text{H}}_{ 2} {\text{O }} + {\text{ xH}}_{ 3} {\text{AsO}}_{ 3} \hfill \\ \end{aligned}$$

Equation (5) coupled with loss of H_2_S would explain why groundwater sulfur concentrations are lower than expected for a seawater-rainwater mixture, why sulfur concentrations are negatively correlated with pH, and why with increasing depth (progressive reduction as Eq. (5) moves to the right) sulfur concentration decreases while As concentration increases (Fig. 8). Furthermore, oxidation of DOC (CH_2_O) would cause a decrease in groundwater Eh, which is significantly lower than in surface water in this area and in seawater [13]. Reaction path modeling [35] and previous reports [36] support our interpretation that progressive reaction of DOC during burial extends through phases of HFO reduction, As mobilization, and sulfate reduction.

For quantitative comparisons of groundwater compositions in the dry season in May and the wet season in October we ran paired t-tests, or if assumptions for the parametric test were not met we ran the Wilconox Signed Rank Test in Sigmaplot v. 12. Bootstrap calculations in SPSS v. 23 to correct for multiple comparisons effects yielded similar results. In May log K, log S, and log Br were higher, while in October pH, SpC, log As, log Fe, log P, log DIC and log DOC were higher. Except for DOC (CH_2_O), concentrations of the reactants in Eq. (5) are higher in May, while the concentrations of products are higher in October (sulfur concentration is assumed to decrease in October through loss of H_2_S). While groundwater temperature is significantly higher in May than October, the difference is only 1 °C, so temperature changes are unlikely to be responsible for this effect. A more likely explanation is that labile DOC is added to the shallow aquifer during the wet season, when rice agriculture is active and organic fertilizers are added to the soil, causing the reaction in Eq. (5) to shift to the right. This is consistent with work showing that application of cow dung to rice paddies in Bangladesh raised porewater DOC and As concentrations [46].

Like this study, the Bangladesh National Hydrochemical Survey of 1998–1999 found limited variation of As concentration with time and no support for the “pyrite oxidation” hypothesis for As mobilization [16]. The lack of correlation of concentrations of Fe and dissolved As was also observed for groundwaters in Araihazar, Bangladesh [29]. A study in the Mekong river delta showed that organic matter in sediments causes reduction of HFOs and increase in dissolved As, and that continued reduction leads to sulfate reduction and sulfide removal, as observed in this study [36].

## Conclusions

All sampled groundwaters in Polder 32 of SW Bangladesh could have formed as mixtures of seawater and freshwater, similar in composition to modern tidal water, followed by progressive reduction causing changes in concentrations of some redox species, and variable amounts of recharge by modern freshwater. Discontinuous silt-mud layers in Holocene aquifer sediments cause anisotropy in permeability and flow, which results in groundwaters having highly variable salinities, As concentrations and ages. Groundwater from tubewells has high DOC comparable to modern porewaters in the adjacent Sundarbans [17] that could have caused reductive dissolution of HFOs and mobilization of As. Seasonal changes in groundwater composition suggest that introduction of labile organic carbon in the wet season in which rice agriculture is practiced may cause HFO reduction and As mobilization. Arsenic concentrations are generally high in the area, with 83 % of our groundwater samples exceeding the WHO guideline of 10 μg/L, and 46 % higher than the Bangladesh standard of 50 μg/L. Furthermore, 100 % of the groundwater samples exceeded the Bangladesh Government salinity guideline of 2 mS/cm. Contamination of groundwater in the shallow aquifer by salts, arsenic and pathogens severely restricts options for potable water sources in the area and may require treatment solutions or import of safe water.
